# Impacts of the Residual Trace Metals of Aquaculture in Net Cages on the Quality of Sediment

**DOI:** 10.3390/life13020338

**Published:** 2023-01-27

**Authors:** Hênio do Nascimento Melo Júnior, Francisco José de Paula Filho, Jorge Marcel Coelho Menezes, José Augusto Soares de Araújo, Jorge Ederson Gonçalves Santana, Hênio Vitor Sobral Melo, Rosimara de Sales Vieira, Cícera Datiane de Morais Oliveira-Tintino, Saulo Relison Tintino, Henrique Douglas Melo Coutinho, Raimundo Nonato Pereira Teixeira

**Affiliations:** 1 Department of Biological Science and Biological Chemistry, Regional University of Cariri (URCA), Crato 63105-000, Brazil; 2 Department of Materials Engineering, Federal University of Cariri (UFCA), Juazeiro do Norte 63048-080, Brazil; 3 Science and Technology Center, State University of Paraíba (UEPB), Campina Grande 58429-500, Brazil

**Keywords:** trace metals, semi-arid, sediment, *Oreochromis*, fish farming, net cage

## Abstract

Anthropogenic pollution by trace metals in aquatic environments in semiarid zones is a critical area of investigation. The objective of this study was to investigate the concentration and spatial distribution of trace metals in surface sediments in the Rosário reservoir, which is affected by the intensive aquaculture of Tilápia-do-Nilo (*Oreochromis niloticus*). Sediment samples were collected in three different areas, postculture (PCTV), cultivation (CTV) and control (CTRL) in the dry season in 2019. The granulometric composition, organic matter and concentrations of Fe, Mn, Zn, Cu, Cr, Cd, Pb and Ni metals were determined. Multivariate statistics were used. Geochemical and ecotoxicological indices and a comparison with sediment quality guidelines (SQG) were used. The sediment was characterized by silty clay loam with an average organic matter of 18.76 ± 4.27. The analytical merit figures demonstrated accuracy (metal recoveries in certified standards) between 89 to 99% and high precision (RSD < 5%). The concentration ranges for the metals were Fe: 0.11–0.85 (%), Mn: 14.46–86.91, Zn: 2.6–220.56, Cu: 26.89–98.75, Cr: 60.18–76.06, Cd: 0.38–0.59, Pb: 18.13–43.13, and Ni: 34.4–46.75, all in (mg/kg^−1^). The highest concentration values were found in the CTV areas (Fe: 40 ± 0.22, Mn: 66.48 ± 19.11, Zn: 114.83 ± 59.75 and Cr: 70.85 ± 2.62) and PCTV (Cd: 0.53 ± 0.04, Cu: 71.83 ± 21.20, Pb: 33.71 ± 4.34 and Ni: 44.60 ± 1.79). Pearson’s correlation, hierarchical cluster analysis and principal component analysis confirmed the influence of fish farming on metals. Only Ni presented concentration values higher than the reference value established in the SQG. Thus, considering the probable geochemical and ecotoxicological effects, they comprise the two lowest levels of impact.

## 1. Introduction

Pollution by trace metals in artificial water reservoirs has attracted worldwide concern [1,2]. Under natural conditions, the increase in trace metal content in the bottom sediments is a result of long-term accumulation from atmospheric deposition and the input of terrigenous materials eroded from the basin [2]. On the other hand, the anthropogenic emissions from human activities that occur in the drainage basin have exceeded natural loads [3]. Retention in reservoir sediments allows accumulated metals to pose a significant threat to ecosystem health [3,4,5].

As the sediment preserves its chemical characteristics over time, it is possible to identify the impacts and their possible origin, differing it from the liquid medium, in which they would quickly be diluted and dissolved [4,6,7,8]. Significant advances in environmental management can be achieved from the assessment of concentrations of metals in sediments, allowing for the determination of pollution sources, locating retention areas and the chronology of metallic pollutants entering a water body [8,9].

Data from the Food and Agriculture Organization of the United Nations FAO [10] indicate that, since 1961, fish consumption has grown by 3% a year and the human population has grown by 1.6%; additionally, aquaculture production is an important source of animal protein and, in 2020, it reached 122.6 million tons, while the production of fisheries continues to decline due to overfishing. The report “State of world fisheries and aquaculture (SOFIA)” by the United Nations Food and Agriculture Organization reported that world aquaculture production grew by an average of 5.3% per year in the period from 2001 to 2018 [10]. 

Brazil appears to be among the main world producers of fish, occupying the 13th place in the ranking. The growth of fish farming between 2014 and 2021 was 45.7%, reaching a production of 841,005 t [11]. Part of this production occurs with the intensive cultivation in net cage of the Tilápia-do-Nilo (*Oreochromis niloticus*) using the water surface of reservoirs intended for multiple uses. This practice was widely disseminated in the Northeast of Brazil, as it presents several public reservoirs.

According to the Municipal Livestock Survey from the Brazilian Institute of Geography and Statistics (IBGE), Brazilian fish farming has grown at an average rate of 4.0% per year since 2017, with the northeast being responsible for 19% of fish production [12]. The expressive participation of intensive pisciculture in net cages, however, is not accompanied by studies on the impacts of residues and effluents from the activity on the sediment of the cultivation areas.

Fish farming in net cages is practiced intensively and has been widely used, resulting in economic benefits as well as serious environmental problems [8,13]. Nutrients, phosphorus and nitrogen loads, increasing eutrophication, the escape of farmed fish to the environment, changes in the behavior of native species and the occurrence of pathogens in wild fish were reported by Phillips, Beverigde and Roos [14].

Other studies have reported the sedimentation of feed residues [15], greater sedimentation in the net cage area [16], an increase in organic matter in the sediment [13], heavy metals sedimentation through feed, antifouling and antirust paints [9,17,18].

Feeds for fish farming have been identified as a source of metals for the sediment: Cu and Zn [19]; Fe, Mn, Zn, Cu, Cd, Cr and Pb [20]; and Cr, Ni, Co, Mn, Cu, Zn, Fe and Mo [21]. Feed as a source of metals for sediment and native fish was observed by Anim-Gayampo, Kumi and Zango [22] in the Tono Reservoir in Ghana and Xie et al. [18] in the Changshou Reservoir, China.

Heavy metals include essential (Cu, Zn, Cr, Mn and Fe) and nonessential elements (Cd, Pb, As, Ni and Hg), and they are important in ecotoxicological studies due to their toxicity, persistence, bioaccumulation and biomagnification in the food chain [23,24].

There is extensive literature related to the accumulation of trace metals in sediments and fish [1,21,25,26,27,28,29,30,31,32,33]. The most usual analytical methods are performed by extracting metals from the sediment by acid combustion and analysis by flame atomic absorption spectrophotometry [34] or by inductively coupled plasma optical emission spectrometry [3,35].

Research that addresses the impacts on sediment caused by fish farming in net cage began due to the expansion of this activity in marine fish farming [36,37,38] and in lake fish farming [13,34,39,40].

Total dependence on fish feed results in residual loads that tend to accumulate in the sediment [14] characterizing the strong relationship of net cage fish farming with sediment.

Studies have shown the relationship between fish farming in net cages and sediment, demonstrated under the following conditions: the sedimentation of feed residues [15]; greater sedimentation in the net cage area [16]; an increment in organic matter in the sediment [13]; and heavy metals in feed, antifouling and antirust paints [9,17,18].

Karaouzas et al. [41] observed different results between concentrations of metals in water and sediment in 68 lakes in Greece. The water samples were classified as good quality. Most sediment exceeded the quality guideline, and in 9 rivers and 3 lakes, it was classified as moderately or highly contaminated.

In studies on sediment contamination, Varol [7], Kalantzi et al. [42], Xie et al. [18] and Wang et al. [8] analyzed the surface sediment, a layer between 0 and 10 cm, which represents a sedimentation time scale. These studies are usually carried out with a single collection that represents a temporal scale of sedimentation, as performed by Wenchuan et al. [43], Hardaway et al. [35], Braga et al. [44] and Gu et al. [45].

The sediment quality guideline is the most commonly used method to assess sediment contamination. This method has been used in several regions of the world; for example, it has been used in the Brazilian Guidelines based on Resolution 454/2012—National Council for the Environment [46]; ICSQG—Interim Canadian Sediment Quality Guidelines; SQGs—Macdonald et al. [47]; EPA—Taiwan [48]; and Australian and New Zealand Guidelines [49].

The application of geochemical and ecotoxicological indices are important tools for the assessment of sediment quality and have been used in research related to the impacts of aquaculture activity on sediments [9,13,17,18,50,51].

The geoaccumulation index [52], the enrichment factor [53] and the contamination factor [54] are individual indices and are calculated from the metal concentration in the environment, the background value or literature reference [55]. Complex indices use the sum of concentrations and, in some cases, use values from other indices [55]. Among the complex or multielementary indices, the most used are the degree of contamination [54]; ecological risk [54] and potential ecological risk [56].

Studies have demonstrated the occurrence of the contamination of sediments by trace metals caused by human activities, urbanization and industrialization [43,57,58,59,60]; agriculture [61,62]; and mining [63,64,65].

Komala et al. [3] stated that several human activities, including fish farming in net cages, impact sediment more than water. Varol et al. [7] showed that sediments are more impacted by fish farms than the water column and, therefore, suggest that sediment monitoring programs be implemented to ensure the sustainable use of reservoirs.

This research aims to increase the knowledge about the impacts of fish farming in net cages on sediment. In this study, the Rosário reservoir, where there was a fish farm in a net cage from 2007 to 2019, was investigated with the following objectives: to understand the effects of the long cultivation period on the sediment, describe the spatial distribution of residual trace metals from fish farming and identify the quality of the sediment using geochemical and ecotoxicological indices.

## 2. Materials and Methods

### 2.1. Study Area

**Figure 1:**
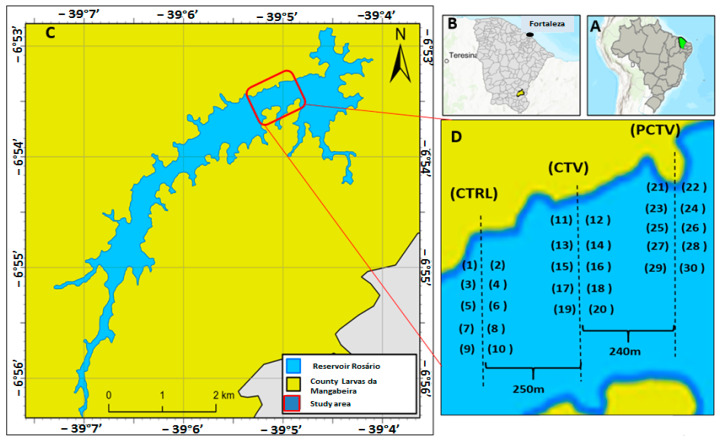
Location of the Rosário reservoir and distribution of collection points, postculture (PCTV), fish farming, culture (CTV) and control (CTRL). (**A**) Brazil, (**B**) Ceará (**C**) reservoir Rosário and (**D**) study area.

The Rosário reservoir is located in the Brazilian semiarid region, Ceará (6°53′24.70″ S/39°4′52.15″ W), is 7.1 km long, has a water volume of 47.22 hm3 and is inserted in the hydrographic basin of the Salgado River with an area drained from 13,450.94 km^2^ (6°00′ to 7°50′ S and 38°30′ to 39°45′ W) (Figure 1). According to the classification of the National Water Agency (ANA) in 2020, the reservoir is classified as small because it has a water volume (m^3^) between 106 and 108.

Its drainage basin covers an area of 336.97 km^2^, with a low population density and incipient economic activity in its surroundings, mainly focused on subsistence agriculture and livestock. The reservoir has multiple uses serving animal watering, balneary and recreation, irrigation, artisanal fisheries, agriculture and fish farming [66,67]. The region’s climate has high temperatures and low annual thermal amplitudes with marked rainfall irregularity and an average of 700 mm yr^−1^ and is markedly subject to rainfall irregularity. The area is mostly occupied by vegetation of the caatinga type (71%) and humid forest (4%), exposed soil (24%), urban area (0.3%) rivers and flooded areas (0.7%). Four soil classes predominate in the area: Latosol, Podzolic, Alluvial and Lithic [68].

### 2.2. Fish Farming in the Study Area

The fish farming activity in net cages was carried out in the reservoir between 2007 and 2019. The cultivated species was tilapia (*Oreochromis niloticus* Linnaeus, 1758). The net cages were 8 m^3^ and had an initial density of 125 fish/m^3^ (1000/8 m^3^). The use of good management practices reduced the density to 31.25 fish/m^3^ (250/8 m^3^) cultivated in approximately 135 net cages distributed in an area of 2.8 hectares of water mirror. The average monthly and annual production was 10.5 t/month and 126 t/year, respectively. The average feed conversion rate was 1.7 kg of feed for 1 kg of fish.

The activity suffered strongly from extreme weather events between 2011 and 2019, related to the most severe and prolonged drought recorded in the northeast region of Brazil in the last 100 years. For six consecutive years (2012–2017), rainfall was up to 50% below the average in the region (700 mm), affecting the water recharge of aquifers, lakes and reservoirs, leading to the depreciation of the hydrogeochemical conditions of water quality. Climatological, meteorological and limnological adversities related to the prolonged period of drought in the Brazilian semiarid region triggered deleterious impacts on the aquaculture activity carried out in the Rosário reservoir. Turbulent vertical circulation events caused serious economic damage to fish farmers, which determined the closure of activities in 2019 [69].

The collection areas were determined in three transects called postculture (PCTC), fish culture (CTV) and control (CTRL). In each transect, duplicates of samples were collected at five different points (Figure 1).

The collection areas in the sampling period had depths varying between 9.0 and 12.0 m. The control area (CTRL—control area) had a preserved riparian forest, and with rare human intervention, low-intensity artisanal fishing occasionally occurs. The area of fish farming in net cages (CTV—fish-farming area) was under the influence of fish farming. The postculture area (PCTV—non-fish-farming area) had the highest number of anthropic activities: commercial fishing activity for *Macrobrachium jelski* and *Macrobrachium amazonico* shrimp. This last area was used as a reference to evaluate the possibility of the dispersion of residual metals from the crop.

### 2.3. Collection, Preparation and Analysis of Sediment

The surface sediment collection was carried out in duplicate in the summer of 2019, which, according to Cortez, Lima and Sakamoto [70], also corresponded to the rainy season of 2019. The first 10 cm of depth were considered as representing the stratum most influenced by physical, chemical and biological phenomena in the sedimentary compartment [7,8,18,42].

The sediment was collected using a kajak collector and was placed in a thermal box and taken to the Limnology and Aquaculture laboratory at the Regional University of Cariri—URCA. All the materials used were previously washed with a 10% HCl solution and rinsed with distilled water.

The sediment was stored at 4 °C until sample processing. After drying, it was crushed and separated in a 2 mm mesh sieve. The sediment texture analysis was performed using a granulometric test for fine materials according to EMBRAPA (2012). The organic matter content was estimated by heating samples in a muffle furnace at 550 °C for 5 h.

The partial acid extraction of metals used 2.0 g of sediment, submitted to acid digestion in 25 mL of aqua regia solution (3HCl/1HNO_3_) 50% *v*/*v*. Acid extraction was performed in a thermokinetic reactor with recirculation in a closed system at 80 °C for 2 h. After extraction, the samples were centrifuged at 3000 rpm/10 min. The analyses were carried out with the Varian/Agilent flame atomic absorption spectrophotometer, model EspectAA 50 B, at the Central Analytical Laboratory of the Federal University of Cariri. The control and quality assurance of the analyses was assessed through the parallel analysis of a certified sediment sample from the National Institute of Standards and Technology (NIST 1646th) (Table 1). Certified standards for the analyzed metals were used in the construction of the analytical calibration curves. Detection limits were calculated following the methodology determined by USEPA (2000). All samples were tested in duplicate. The relative standard deviation (RSD) was smaller than 5%.

### 2.4. Geostatistical Assessment

The analyzed data were organized using the WPS Spreadsheets electronic spreadsheet and statistically processed with the PAST 4.03 software: Paleontological Statistics Software Package for Education and Data Analysis.

The spatial distribution of trace metals was expressed with nonparameterized data. The normalization of the data was verified with the Lilliefors test and, when necessary, the logarithmic transformation was performed. A one-way analysis of variance (ANOVA) was used to compare data averages from the studied areas.

Multivariate analysis of the sediment, comprising Pearson’s correlation (*p* < 0.05), was used to assess pairs of variables and improve understanding of the relationships between trace metals and the environment. The principal components analysis (PCA) was preceded by the sphericity tests of Bartlett and Kaiser–Meyer–Olkin, the varimax rotation was used to optimize the variance and, finally, the hierarchical cluster analysis (HCA) was performed using the Ward method to verify similarity across the Euclidean distance.

### 2.5. Geochemical and Ecotoxicological Indices

To assess the risks of sediment contamination, geochemical indices (Table 2) and ecotoxicological indices (Table 3) were used. Due to the lack of background value and/or any reference value, the attempt to use data from the literature generated incongruous results.

The application of geochemical indices occurred, according to Caeiro et al. [57], based on reference points of the environment, which were areas with the greatest preservation and least human intervention, and the baseline for application in the geochemical indices was determined.

The ecotoxicological indices, sediment quality guidelines (DQS) and toxic risk index were carried out in accordance with Resolution No. sediment from the Canadian Council of Minister of the Environment (CCME) [71] and National Oceanic and Atmospheric and Administration (NOAA).

## 3. Results and Discussion

### 3.1. Sediment Constitution

The granulometric analysis showed that the sediment had a silty clay–loam constitution with the respective percentage variations: sand: 5.05% to 33.93% and an average of 14.48 ± 7.94; clay: 4.67% to 80.81% and an average of 35.88 ± 21.71; and silt: 5.32% to 90.29% with an average of 50.54 ± 23.34. For the analyzed areas, it was verified that the percentages presented decreasing average values in the order of sand, clay and silt (Table 4).

**Figure 2:**
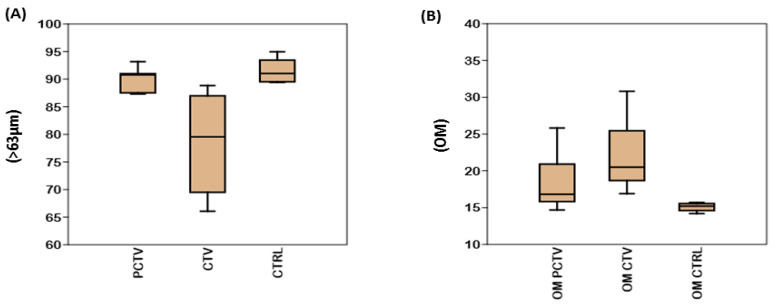
Spatial distribution of the superficial sediment of the Rosário reservoir. (**A**) Percentage of silt/clay mineral fraction (<63 µm). (**B**) Percentage of organic matter.

The distribution of the fraction <63 µm, silt/clay, in descending order, corresponded to CTRL, PCTV and CTV. The analysis of variance of the fraction, <63 µm of the PCTV area in relation to CTV and CTRL, resulted in (*p* < 0.01). However, between CTV and CTRL, there was no statistically significant difference (*p* > 0.05) (Figure 2A).

Martins and Souza [72] analyzed alluvial deposits granulometry in the Saco creek drainage basin in the semiarid region of Pernambuco State, Brazil, and they verified the presence of cambisols and clayey soils; additionally, they found that the variation in the silt clay fraction was 82.6% upstream and 76.3% downstream of the studied environment area.

Similar to the aforementioned study, the Rosário reservoir watershed is in an area with eutrophic red clay soil, and the variation in the silt clay fraction in the three cultivation areas corresponded to 87.30% (PCTV), 81.37% (CTV) and 88.58% (CTRL).

High values of the silt/clay fraction were also found by Alpaslan and Pulatsü [73] in the Kesikköprü Turkia reservoir for fish farming (95.60%) and the control (90.26%).

Karikari et al. [13] reported that sediments with larger particles have a greater possibility of resilience. These authors determined the sediment texture of Lake Volta in Ghana in the fish farming area with 57.3% sand, 20.0% silt and 22.5% clay, and the reference area with 60.4% sand, 18.8% silt and 20.8% clay.

The organic matter (OM) present in the sediment of the Rosário reservoir varied between 14.20% and 30.80%, with an average percentage of 18.76 ± 4.27. The three studied areas resulted, respectively, in average percentages of 21.95 ± 4.40 in the cultivation (CTV), 18.49 ± 3.60 in the postculture (PTCV) and 15.84 ± 2.39 in the control (CTRL).

The ANOVA test showed that there was no significance between PCTV and CTV (*p* > 0.05), but the control area was statistically different from the other areas with (*p* < 0.01) for the PCTV and CTV areas (Figure 2B).

Contamination by organic matter in sediment can be classified into three levels: uncontaminated sediment with 0.5% to 5.0%; slightly contaminated with 5.0% to 15.0% and typically contaminated with 15% or more [13,74,75,76].

The results obtained in this study suggest that the Rosário reservoir is typically contaminated; however, in addition to fish farming, there was a source of autochthonous organic matter, and the vegetation cover of the reservoir area was not removed before the flooding. Therefore, fish farming and vegetation cover that is not removed correspond to these results, especially because the reservoir is in an area characterized exclusively by small-scale agricultural activities.

The organic matter in the sediment of fish farming areas in net cages, in addition to the intensity and technology of cultivation, are also related to the natural conditions of the environments and to the human activities carried out in the hydrographic basin.

The changes in organic matter, promoted in 12 years of cultivation, were not enough to make the CTV and PCTV areas significantly different; therefore, they are statistically similar in terms of organic matter content in the sediments.

The control area had a significant difference in the percentage of cultivation and postculture areas, which were 6.11% and 2.65%, respectively, which is consistent with the values reported by Karikari et al. [13] for Lake Volta, which had a variation of 4.42–8.98% in fish farming and 6.5% to 12% in the reference area.

The annual production of tilapia in Lake Volta (34,692 to 76,845 t/year) was lower than the production of the Rosário reservoir (126 t/year), meaning that the productive intensity is probably one of the main determining factors of the observed difference.

The production of trout in the Kesikköprü reservoir, Peru, corresponded to 20 t/year with a variation of 13.12% to 15.57% of organic matter in the cultivated area [73].

In superior proportions to our study, regarding the production of trout in Lake Passage, Canada, Cornel and Whoriskey [77] identified that the organic matter content of the sediment in the fish farming area ranged from 39% to 69% for a production of 14 t/year with a feed conversion ratio of 3.7:1.

The comparison of the above data indicates that the adopted technological package possibly exerts influence on the residual organic loads. The distribution of organic matter, with a higher percentage in the cultivated area, may be an indication that fish farming is the main source of organic matter among the areas studied.

### 3.2. Concentration and Distribution of Trace Metals in the Sediment

**Figure 3:**
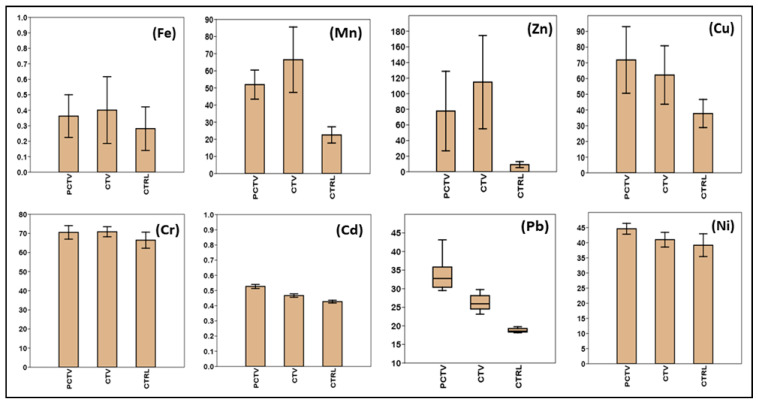
Concentrations of trace metals in the superficial sediment of the Rosário reservoir. Non-fish-farming area (PCTV), fish farming area (CTV) and control area (CTRL).

The concentrations of trace metals in the sediment analyzed showed that fish farming activities caused an increase in concentrations in the cultivation area (CTV) and postculture area (PCTV). In these two environments, higher concentrations were observed than in the control area (CTRL) (Figure 3).

The control area (CTRL) represents the natural characteristics of the studied environment as it corresponds to the area of less human intervention; therefore, it is where the lowest average values of concentrations of the metals were verified. The decreasing order of metal concentrations occurred in the following order: Fe > Cr > Ni > Cu > Mn > Pb > Zn > Cd.

In the fish farming area (CTV), the highest average values of the main trace metals present in the composition of the tilapia feed, Fe, Mn, Zn and Cr, were observed (Table 5). The decreasing order of metals in the fish farming area was Fe > Zn > Cr > Mn > Cu > Ni > Pb > Cd.

In the postculture environment (PCTV), the point of analysis to verify the dispersion of residual loads from fish farming had the highest concentrations of the metals Cd, Cu, Pb and Ni (Table 5). In this area, the following decreasing order was observed: Fe > Zn > Cu > Cr > Mn > Ni > Pb > Cd.

In the Gokcekaya Reservoir, Turkia, Karakoca and Topcu [78] identified that the sediment from the rainbow trout production areas showed the highest concentrations of the most significant metals in the feed, Fe > Mn > Ni > Zn > Cr > Cu.

Similarly, the results of this study also revealed that in the cultivation and postculture areas of the Rosário reservoir, the highest concentrations of metals correspond to the main trace metals in the tilapia feed, Fe, Zn, Mn, Cu and Cr.

Magna et al. [79] evaluated trace metals in tissues of tilapia cultured in net cages in the Volta River basin, Ghana, Lake Volta (fish farms A and B) and River Volta (fish farm C). Tilapia tissue concentrations were classified as follows: Fe > Mn > Zn > Ni > Cr (for fish farm A), Fe > Zn > Ni > Mn (for fish farm B) and Fe > Mn > Zn > Ni > Cr. However, these authors found that the concentrations of metals in the tissues do not imply danger for human consumption.

Oliveira et al. [19] analyzed fish, sediment and fish feed in the Castanhão reservoir, semiarid region of Brazil, and observed that only 0.13% of Mn and 2.61% of Zn are incorporated by cultivated tilapia, that Cu absorption is 47.4% and that the excess percentage of these metals are sedimented and/or dispersed to other areas of the environment. The results pointed out by Oliveira et al. [19] are indicators of the concentrations and dispersion of trace metals verified in the Rosário reservoir, occurring between the fish farming area (CTV) and the postculture area (PCTV).

However, a study carried out by Varol [7] showed that in fish farming in the Kaban reservoir, Turkey, the concentrations of residual metals in the feed, Fe, Mn, Zn, Cu, Co and Cd, decreased with the distance from fish farming.

### 3.3. Percentage Increase in Trace Metal Concentrations in the Sediment

The concentrations of trace metals in the CTV and PCTV areas showed increases in concentrations compared to the CTRL area. In the cultivation area, the highest percentage growths were observed for the metals Zn, Mn, Cu and Pb, and in the postculture area, the highest percentage growth values were for the metals Zn, Fe, Pb and Cu (Table 6).

Cd was the only metal that was an exception to this increase, and the control area had the highest percentage value in relation to the cultivation and postculture areas, which were 12.77% and 23.26%, respectively.

Karikari et al. [13] found that the sediment of the fish farming area in relation to the reference area had the following percentage growth: Pb: 38.38%; Zn: 68.05%; Fe: 98.08%; Mn: 0.16%; and Se: 65.38%. The growth of Cu was verified in the reference area in relation to the cultivation corresponding to 88.37%.

In addition to the increase verified during the cultivation period in the Rosário reservoir, we can estimate the possibility of the future accumulation of residual metals from fish farming according to Oliveira et al. [19]. For these authors, the technological package of tilapia production in the semiarid region of Brazil is predominantly standardized both in terms of cultivation management and the quality of the feed for tilapia cultivation. The authors point out that the Castanhão reservoir model can be extrapolated to other fish farms in the northeast region. In this sense, if the fish farming in the Rosário reservoir had continued its annual production of 126 tons, the annual residual loads of metals from the feed to the sediment would be around 0.38 kg of Zn, 0.03 kg of Cu and 0.07 kg of Mn.

As for the future trend of the accumulation of metals in the sediment of reservoirs on a macro scale covering the entire Brazilian territory, the data produced by BR Peixe (2022) indicate that growth in Brazilian fish farming from 2014 to 2021 is in the order of 5.6% per year. This may be an indication of the possibility of a future increase in residual loads from fish farming activities in Brazil.

### 3.4. Sediment Quality Guidelines (SQGs)

The SQGs criteria of Resolution No. 454/2012-CONAMA, criteria N1—Threshold below which there is a lower probability of adverse effects to the biota and N2—threshold above which there is a greater probability of adverse effects to the biota correspond to international legislation: TEC: threshold effect concentration; PEC: probable effect concentration [47], respectively.

The comparison of trace metal concentrations in the Rosário reservoir sediment with the sediment quality criteria [46] indicated that most metals have concentrations that are distributed in the range of the legal limits determined for Brazil (Table 6).

Fe and Mn are not included in the standardization of sediment quality guidelines. Cd had all concentrations below the limits determined in the SQGs. Zn, Cu, Cd and Pb had concentrations below the N1 criterion. Among the criteria N1 and N2, concentrations of Zn, Cu and PB were obtained. Cr exclusively presented concentrations distributed within the range of the legal threshold, criteria N1 and N2 (Table 6).

Among the metals analyzed in the sediment of the Rosário reservoir, only nickel had 90% of its concentrations above the established limit. Xie et al. [18] reported a similar situation in the Changshou reservoir, where nickel had 86% concentrations above the maximum limit of the sediment quality guidelines.

The comparison of the concentrations of metals in the sediment of the Rosário reservoir with the SQGs CONAMA [46] resulted in the following conditions: concentrations < N1 (Zn 80%), (Cu 20%), (Cd 100%) and (Pb 90%); concentrations > N1 and < N2 (Zn 20%), (Cu 80%), (Cr 100%), (Pb 10%) and (Ni 10%); concentrations > N2 (Ni 90%).

Xie et al. [18] evaluated the sediment of the Changshou Reservoir and classified the metals Zn, Cr, Cu, Pb and Ni as probable inputs of the fish farming activity. In this study, the sediment quality guideline had results below the limits established for the metals Cu = 29%, Hg = 100% and Pb = 42%, and the values between the limits corresponded to Zn = 100%, Cu = 71 %, Cr = 100%, Ni = 14% and Pb = 58%.

For the SQGs of the sediment from the Rosário reservoir, the results were also similar to the results reported by Xie et al. [18] for the metals Cu and Cr.

In the Magobo Reservoir, Zimbabwe, Kanda et al. [50] found that all concentrations of toxic trace elements were below the threshold effect-TEL/minimum threshold for freshwater ecosystems, suggesting that there are no obvious adverse biological effects.

Varol [7] evaluated the concentrations of metals in the sediment of three fish farming areas in net cages in Lake Keban, Turkey, to determine the sediment quality guidelines and found that the metals Cd, Cu, Pb and Zn were below the established limits of the TEC: threshold effect concentration and PEC: probable effect concentration.

### 3.5. Increase in Metals Trace of Ctv and Pctv Areas in Relation to CTRL

The comparison between the averages of the areas, carried out using a one-way ANOVA test, confirmed that there was a statistically significant difference between the analyzed areas, proving that there was an increase in the concentrations of metals from the cultivation area.

The analysis of variance showed that there was a significant difference between the CTRL and CTV areas for the metals Mn, Zn, Cu and Cd (*p* < 0.01), as well as Cr and Pb (*p* < 0.05). For Ni, there was no significant difference.

A comparison of the CTRL and PCTV area resulted in a difference for the metals Mn, Zn, Cu, Cd and Ni (*p* < 0.01) and Cr and Pb (*p* < 0.05).

However, between the CTV and PCTV areas, the metals Mn, Zn, Cu and Cr were statistically similar (*p* > 0.05). However, significant differences were identified for the metals Cd and Pb (*p* < 0.05) and Ni (*p* < 0.01).

Fe was an exception in all tests, as it was revealed that there was similarity between the analyzed areas (*p* > 0.05).

Cacho, Moura and Henry-Silva [80] compared the sedimentation rate of fish farming with that in a reference area of the Umari reservoir, RN, Brazil. The Kruskal–Wallis tests indicated that there was a significant increase in sedimentation in the fish farming area. However, the authors emphasized that these changes were punctual, restricted to the area of fish farming.

The reality observed in the Rosário reservoir sediment was different, and the CTRL area was significantly different from the CTV and PCTV areas; however, between the CTV and PCTV areas, there was significant statistical equality, which suggests that there was a dispersion in the residual loads from the CTV to the PCTV area.

Among the factors that allow for the dispersion of metals from the sediment, pH variations were the most significant [78], as the increase in pH favors the adsorption and precipitation of metals [9] since the release to water can occur by pH decrease [81] and sediment oxygenation [19].

In reservoirs in the semiarid zone of Brazil, vertical circulations of the water mass can promote the oxygenation of the sediment. Oliveira et al. [19] warn that in periods of drought, the reduction in water volume reduces the size of the water column and allows for better oxygenation of the sediment.

Henry-Silva, Melo-Júnior and Attayde [82] reported that in the Umari Reservoir, RN, Brazil, a heavy summer rain event cooled the water temperature, breaking the thermal stratification and causing turbulent vertical circulation. This event triggered sediment suspension and the complete mixing of the water column.

In the Rosário reservoir, vertical circulation processes occur daily, and the high temperatures during the day and the cooling during the night promote vertical circulation, which allows for the oxygenation of the reducing zone and the surface sediment.

Melo-Júnior and Campeche [69] and Melo-Júnior and Sampaio [83] warned that, in the Brazilian semiarid region, the transition between summer and winter has greater thermal variations and more intense winds that increase vertical circulation and can also cause turbulent vertical circulation. These processes act by allowing for the oxygenation of the sediment, as well as allowing for the dispersion of the sediment.

### 3.6. Patterns of Inter-relationships between Organic Matter and Metal Residual Traces of Pisicultutra

Pearson’s correlation showed that the fraction <63 μm had a nonsignificant correlation (*p* > 0.05) with all metals and with organic matter, and that the r-Pearson values ranged between 0.0 and 0.36, meaning that it was classified as an insignificant correlation (Table 7).

Organic matter showed statistically significant correlations between *p* < 0.05 and *p* < 0.01 for the metals Mn, Zn, Cu, Cr and Cd, as well as nonsignificant correlations with Fe and Ni and a fraction <63 μm. The r-Pearson values were categorized as insignificant (0.0 to 0.3—Fe and Ni), low (0.3 to 0.5—Zn, Cu, Cr and <63 μm) and moderate (0.5 to 0.7—Mn).

Cd showed a negative correlation with all other metals and with the fraction <63 μm, and in most cases, the r-Pearson ranged between an insignificant (0.0 to 0.3) and low correlation (0.3 to 0.5).

Statistically significant correlations with r-Paerson values classified between moderate (0.5 to 0.7) and high (0.7 to 0.9) were observed in the inter-relationships between the metals: Mn and Zn (<0.01 and 0.75); Cu and Cr (<0.01 and 0.68); Mn and MO (<0.01 and 0.65); Mn and Cu (<0.01 and 0.62); Mn and Cr (<0.01 and 0.56); Zn and Cr (<0.01 and 0.51); and Cu and Cd (<0.01 and −0.51). The correlation with r = 0.41, which corresponds to a low correlation, and with significance *p*<0.05 was verified between Zn and Fe.

Correlations varying between insignificant (0.0 to 0.3) and low (0.3 to 5.0) were observed between Fe and Ni with the other metals and with the sediment, fraction <63 μm and MO.

The correlations of Ni with Cu, Cd and Cr were the only significant ones (*p* < 0.01), as all others resulted in *p* > 0.05.

The results obtained allow us to infer that there was an influence of fish farming on this reality. Pearson’s correlation showed that organic matter has a stronger and more significant correlation with the trace metals present in the fish feed formulation (Mn, Zn, Cr and Cu), and it was also among these metals that the strongest correlations occurred, regarding the significance and r-Pearson value.

As was verified in the sediment of the Rosário reservoir, Karakoca and Topcu [84] found that the strongest correlations of metals in the sediment of Lake Gokcekaya, Turkia occurred in the fish farming area.

Regarding the input of residual metals from culture to the environment, Oliveira et al. [19] reported that the metabolic absorption rate of tilapia cultured in net cages at Castanhão weir, Brazil, corresponded to the percentage of metabolic absorption in the order of 0.13% for Mn, 2.61% for Zn and 47.4% for Cu. Therefore, the remaining percentage constitutes part of the residual organic load released into the environment.

As demonstrated by Montanhini Neto and Ostrensky [85], for the production of 1000 kg of tilapia, 1043 kg of organic matter is released into the environment.

The residual loads of organic matter from fish farming in net cages justify the results verified in our study, and they can explain the spatial distribution of metals and the strong and significant correlations between organic matter and metals that occur more strongly in the fish farming area.

The importance of residual organic matter from fish farming can be understood considering Paula-Filho et al. [86] when they stated that the correlations between organic matter and metals can represent the greater retention of metals by biogenic fractions of the sediment. Komala et al. [3] attributed organic matter as the correlation factor between metals and sediments from fish farming in Lake Manijau, Indonesia.

Karikari et al. [13] suggested that in the Volta-Ghana lake fish farm, the significant differences in the concentrations of metals in the fish farm sediment compared to the control environment can be attributed to organic matter and sediment organic carbon that were higher in volume and statistically different from the control area.

### 3.7. Geostatistical Analysis

The hierarchical cluster analysis resulted in the formation of two groups, an exclusive group representing the CTRL area and the second group formed by two subgroups, represented by the PCTV and CTV areas.

The exclusive formation of the CTRL group is justified by the aspect of this area having its natural characteristics preserved and the inexistence of anthropic activities. The percentages of organic matter and the spatial distribution of trace metals in the sediment, subjected to an ANOVA, corroborate this distinction shown in the cluster analysis.

The stability of the geochemical conditions in the area made it possible for the greatest similarity to have occurred in the CTRL area.

**Figure 4:**
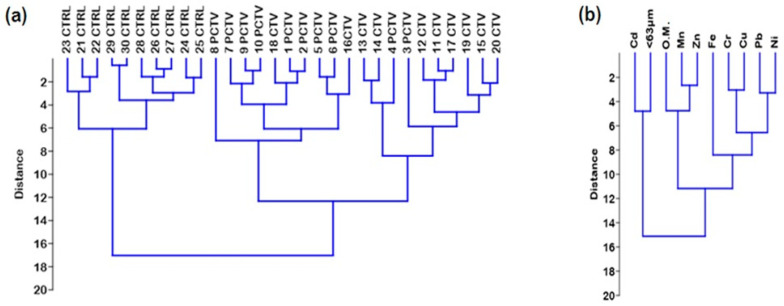
Hierarchical grouping analysis of the studied areas (**a**) and trace metals in the sediment (**b**).

The second group was formed by the PCTV and CTV subgroups that were grouped according to the influence of the CTV area on the environment. The statistical similarity was verified between these two areas, and the significant difference for the control area confirms the configuration expressed in the cluster elaborated for the areas (Figure 4).

In these two groups, it is possible to observe the intersection of similar points but from different groups; in the PCTV subgroup, the points 16CTV and 18CTV are inserted, and in the CTV subgroup, the points 3PCTV and 4PCTV are inserted (Figure 4A). In this group, the greatest Euclidean distance was equivalent to 12.0 and the subgroups had the following distances: PCTV, 7.0; CTV, 8.0.

The factors that determined this grouping in the cluster were the cultivation management and its residual loads acting on the sediment, as well as the events of dispersion of trace metals from the CTV area to the PCTV area. These events made it possible for these two areas to be significantly similar.

According to Henry-Silva, Melo-Júnior and Attayde [82], Melo-Júnior and Campeche [69] and Melo-Júnior and Sampaio [83], vertical circulation processes are common in semiarid reservoirs. Circulation events allow the oxygenation of the sediment which contributes to the dispersion of trace metals in the environment.

Yang et al. [87] reported that the concentrations of metals in the sediment of Lake Daechung, Korea, were obtained in a condition of vertical circulation with oxidized sediment but stated that, certainly, the samples collected in the stratification period with hypoxic conditions would cause the results to be quite different [87].

This is probably the event that determines the dispersion of residual loads from the crop and the similarity between the crop and postcrop areas. Even the insertion verified in the cluster may be a consequence of the phenomena discussed here.

The fact that the cluster showed the CTRL area isolated from the others, as well as the statistical difference for the CTV and PCTV areas, shows that the CTRL area is totally free from the influence of the crop.

The greatest similarities were found between the following points: 30CTRL-29CTRL, 27CTRL-26CTRL, 9PCTV-10PCTV, 1PCTV-2PCTV and 11CTV-12CTV.

The distribution and similarity between the metals and the sediment resulted in the formation of two groups. The first group was formed by Cd and the silt/clay fraction (<63 µm). The second group formed two subgroups: the first with organic matter, Mn and Zn, and the second with the metals Fe, Cr, Cu, Pb and Ni, which are also indirectly linked to organic matter (Figure 4B).

The smallest Euclidean distances were observed in the subgroups of metals directly associated with organic matter, Mn and Zn (≅2.5), and indirectly associated metals, Fe, Cr, Cu, Pb and Ni (≅3.0). This resulting similarity suggests a greater influence of residual organic matter from cultivation on these trace metals.

Cd and the fraction <63 µm formed an isolated group, a fact justified by the predominance of Cd in the control area, where the lowest percentage of organic matter and the highest percentage of the fraction >63 µm were verified.

The hierarchical cluster analysis confirmed the distribution and inter-relationships between the sediment and the metals found between the studied areas of the Rosário reservoir.

The metal cluster showed the weak correlation of the mineral fraction <63 µm and Cd with the other metals and organic matter, separating it into a distinct group. This configuration confirms the Pearson correlation, the fraction <63 µm with little influence on trace metals and Cd with a negative correlation with organic matter.

Another aspect similar to Pearson’s correlation is the grouping formed between organic matter, Mn and Zn. The strongest correlations occurred between Mn and Zn, Mn and organic matter and, at a second level, between Zn and organic matter. This reality is consistent with the cluster, especially because the shortest Euclidean distance was exposed between Mn and Zn.

In a similar way, Chou et al. [88] verified in a fish farming area that the organic fraction, CO, was more grouped to the metals Zn, Mn and Cu than the mineral fraction, >63 µm.

A principal component analysis (PCA) that was preceded by Bartlett’s sphericity test (*p* < 0.05) and the Kaiser–Meyer–Olkin test (0.75) showed that the normalized data were adequate for principal component analysis. In this test, the analyzed metals were explained in three main components, all with an eigenvalue >1 and accounted for 70.72% of the total variance.

The first component (PC1) represented 44.18% of the variance and presented positive loads in Fe, Mn, Zn, Cu, Cr, Pb, Ni and organic matter. The second component represented 15.89% of the variance and positive loads in Pb, Ni, Cu, Fe and <63µm. The third component accounted for 10.64% of the total variance and showed positive loads in Pb, Cd, Mn, Zn and Fe.

**Figure 5:**
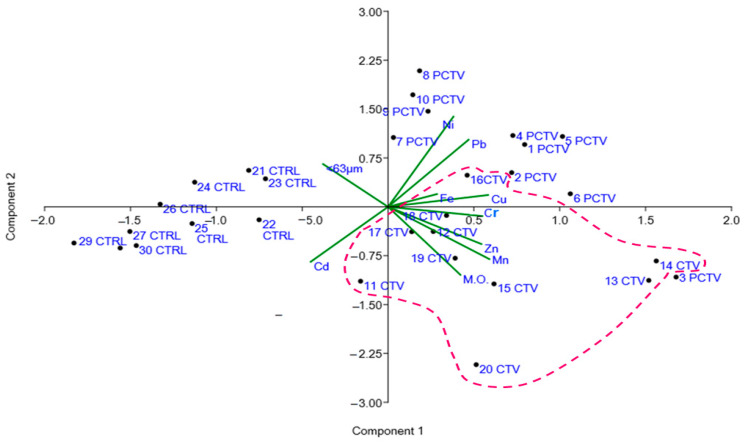
Analysis of the main components of the Rosário reservoir.

The positively charged metals of CP1 were plotted in the same quadrant of the CTV area (Mat. Org., Mn, Zn, Cr, Cu and Fe) and in the quadrant of the PCTV area (Ni and Pb). With negative charges, Cd and <63 µm, which is associated with the CTRL area (Figure 5). This configuration indicates that the residual organic matter from fish farming exerted a greater influence on the metal distribution pattern than the fraction <63 µm.

The principal component analysis was performed to compare the pattern of the distribution of the trace metals in the sediment and corroborate the results of the distribution of metals in the sediment, the correlations, the distinction and similarity between the studied areas, the sediment and the trace metals analyzed.

The metals present in the feed formulation had the highest loads of CP1. The PCA graphic configuration showed that organic matter, Mn, Zn and Cr were equally plotted in the crop area. Fe and Cu were plotted in the area of intersection between cultivation and postcultivation and thus resembled the configuration of the hierarchical cluster analysis, the correlation and distribution of metals.

As shown in the hierarchical cluster analysis, in the postcultivation area, Pb and Ni were plotted, and Cd and <63 µm were antagonistic to the other components of the analysis, being plotted separately.

Multivariate statistics confirmed the influence of organic matter and fish farming on the relationships of trace metals with sediment, reaffirming the biogenic character of the sediment due to fish farming activities.

Chou et al. [88] investigated the effects of aquaculture in Passamoquody Bay, southwest of New Brunswick, Canada. The authors demonstrated that PCA and AHC, associated with the EMP-environmental monitoring program, are excellent for application in net cages fish farming.

In this sense, in regions where net cage fish farming activities are devoid of environmental monitoring programs, multivariate statistical analysis plays an important role in monitoring the activity.

The multivariate statistical analyses used in this study confirmed the spatial distribution of metals in the sediment, evidenced the influence of organic matter on trace metals and showed the influence of the fish farming area (CTV) on the increase in organic matter and metals on the environment. In addition to these aspects, the distribution of metals between the fish farming area (CTV) and postculture area (PCTV) was evidenced.

### 3.8. Geochemical and Ecotoxicological Indexes

#### 3.8.1. Geochemical Indexes

Among the geochemical indices evaluated in this study, all the results were distributed between the initial and intermediate levels of classification. The geoaccumulation (Igeo) and enrichment factor (EF) indices were classified into seven categories. The contamination factor (FC) and the degree of contamination (GC) were classified into four quantitative and qualitative categories.

**Figure 6:**
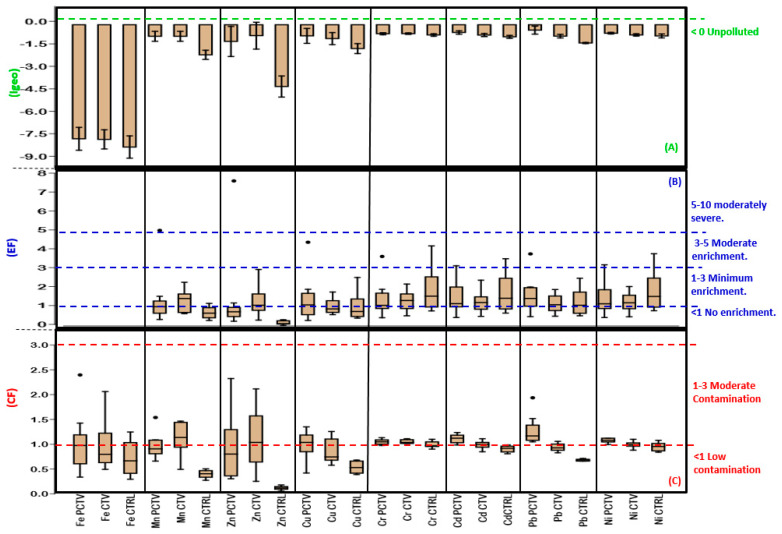
Geoaccumulation index (**A**), enrichment factor (**B**) and contamination factor (**C**) of the superficial sediment of the PCTV, CTV and CTRL areas of the Rosário reservoir (mean ± standard deviation; median, 1°Q, 3°Q and • outlier).

The Igeo results in the three studied areas corresponded to the unpolluted and unpolluted to moderately polluted classes, and in this range, the values varied between 0.54 and −7.55 (Figure 6A).

The ordering of the average Igeo values in the collection areas expressed different orders: PCTV (Pb > Cd > Ni > Cr > Cu > Mn > Zn > Fe), CTV (Mn > Cr > Ni > Cd > Zn > Pb > Cu > Fe) and CTRL (Cr > Ni > Cd > Pb > Cu > Mn > Zn > Fe).

The values of the enrichment factor (EF) of the PCTV, CTV and CRTL areas ranged from 0.02 to 7.45, which corresponded to the following classes: no enrichment, moderate and moderately severe (Figure 6B).

The ordering of the average values of the enrichment factor by areas resulted in the following conditions: PCTV (Zn > Mn > Cu > Pb > Cr > Ni > Cd): 41.43% without enrichment, 48.57% with minimum enrichment, 8.57 % with moderate enrichment and 1.45% with moderately severe enrichment; CTV (Zn > Cd > Mn > Cr > Ni > Cu > Pb): 38.57% without enrichment and 61.43% without minimum enrichment; CTRL (Cr > Ni > Cd > Cu > Pb > Mn > Zn): 55.71% without enrichment, 35.71% without minimum enrichment and 8.57% without moderate enrichment.

The overall result of the contamination factor (CF) in 77% of the cases corresponded to the first classification, low contamination, and 22.92% corresponded to the second category, moderate contamination. Among the three studied areas, the maximum and minimum values observed were, respectively, FC = 2.26 and FC = 0.02. (Figure 6C).

The average values of the contamination factor verified in the three study areas resulted in the following ordinations and classifications: PCTV (Pb > Cd > Ni > Cr > Fe > Cu > Mn > Zn): 58.75% low contamination and 41.25% moderate contamination; CTV (Mn > Zn > Cr > Ni > Cd > Fe > Pb > Cu): 50% low contamination and 22.50% moderate contamination; CTRL (Cr > Ni > Cd > Fe > Pb > Cu > Mn > Zn): 95% low contamination and 5% moderate contamination.

Custodio et al. [51] analyzed two fish farming areas in Peru and identified the contamination factor verified in the Tishgo River: Cu, low contamination; Pb and Zn, moderate contamination; and As, very high contamination. In the Chia River, the following results were found: Cu and Pb, low contamination; Zn, moderate contamination; As, very high contamination.

The general degree of contamination of the Rosário reservoir ranged from 0.83 to 11.80 and was classified into two initial classes, low degree of contamination (CD < 6) and moderate degree of contamination (6 ≤ CD < 12).

**Figure 7:**
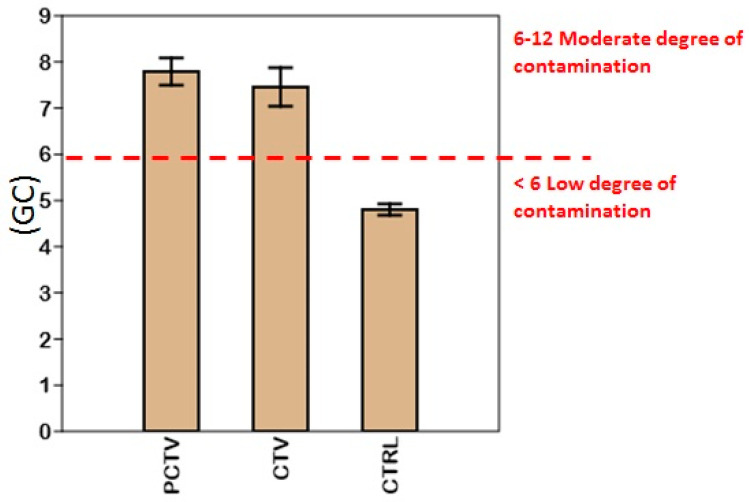
Surface sediment contamination factor of the PCTV, CTV and CTRL areas of the Rosário reservoir (mean ± standard deviation).

The analysis of the degree of contamination by area resulted in a variation between 4.40 and 10.23. PCTV and CTV areas were classified as moderately contaminated and resulted in the following distributions: PCTV (Pb > Cd > Ni > Cr > Fe > Cu > Mn > Zn) and CTV (Mn > Zn > Cr > Ni > Fe > Cd > Pb > Cu). The CTRL area resulted in the following ordering (Cr > Ni > Cd > Fe > Pb > Cu > Mn > Zn) and was classified as having a low degree of contamination (Figure 7).

The results calculated from the geochemical indices of the Rosário reservoir sediment could be corroborated by comparing the concentrations of trace metals with the baseline values of the sediment.

The addition of metals to the sediment in relation to the baseline values resulted in an average increase in the order of 1.2 ± 0.53, an insignificant value that is in agreement with the results of the geochemical indices that corresponded to the lowest classification categories. In the fish farming area in Changshow Lake, China, Xie et al. [18] found that the metals Zn, Fe, Mn, Cu, Ni, Cr, Se, Hg and Pb in the sediment had concentrations above the background value.

The comparison between the averages of the geoaccumulation index revealed that there was no significant difference between the PCTV, CTV and CTRL areas, although the largest accumulations occurred in the PCTV and CTV areas. As for the enrichment factor, a significant difference was found between PCT and CTV.

Regarding the indices of the contamination factor and degree of contamination, it was observed that between PCTV and CTV, there was no significant difference; however, the CTRL area was significantly different from the PCTV and CTV areas.

#### 3.8.2. Ecotoxicological Indexes

The results of the ecotoxicological indices used in this study, i.e., ecological risk ($E_{j}^{i}$), potential ecological risk ($PERI_{j}$) and toxic risk index (TRIi), corresponded to the lowest classification categories.

**Figure 8:**
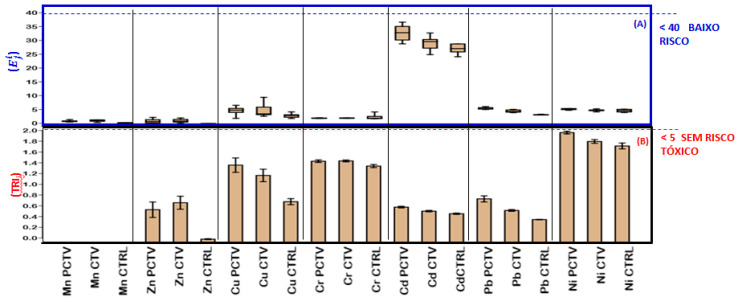
Ecological stream index (**A**) and toxic risk index (**B**) of the surface sediment of the PCTV, CTV and CTRL areas of the Rosário reservoir (median 1st and 3rd (mean ± standard deviation)).

The values of the ecological risk index for the PCTV, CTV and CTRL areas were classified in the first class, low ecological risk ($E_{j}^{i}\;$< 40). Cd was an exception due to the high value of the toxic response coefficient, defined by Hankanson [54], and the calculated values comprised the first and second classes, i.e., low risk ($E_{j}^{i}\;$< 40) and moderate risk (40 <$\;E_{j}^{i}\;$< 80), respectively (Figure 8A).

The values of $E_{j}^{i}$ from the PCTV area presented the following ordering: Cd > Pb > Ni > Cu > Cr > Zn > Mn. In the CTV and CTRL area, the ordering was Cd > Pb > Ni > Cu > Cr > Mn > Zn.

**Figure 9:**
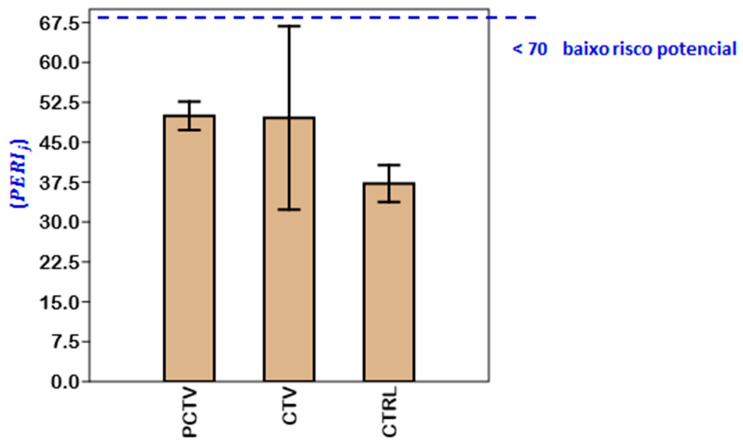
Potential ecological risk of the superficial sediment of the PCTV, CTV and CTRL areas of the Rosário reservoir (mean ± standard deviation).

The potential ecological risk ($PERI_{j}$) for all environments corresponded to first class, low potential risk ($PERI_{j}<70$) (Figure 9).

Possibly, the production intensity of fish farming in the Rosário reservoir has contributed to the potential ecological risk of metals in the sediment being classified as the lowest potential risk.

All the results calculated for the toxic risk index were lower than the first class of this index, with the highest calculated value being 2.5 and the first class corresponding to TRIi ≤5, which is equivalent to no toxic risk (Figure 8B). The comparison between the areas by the Kruskal–Wallis–Dunn test showed that PCTV and CTV were similar (*p* > 0.05). The CTRL area was significantly different from the PCTV and CTV areas (*p* < 0.05).

The results exposed on the Rosário reservoir revealed the minimum condition of toxicological effects on the environment’s biota, and these results are in total consonance with the geochemical indices, as well as with the values of the concentration of metals in the evaluated areas, a fact that reveals the low ability to impact the sediment of the environment.

The calculated results of the ecotoxicological indices of the sediment of the Rosário reservoir can be corroborated by comparing the results of the ecotoxicological indices with the sediment quality guideline. As well, a comparison with the increases in concentrations in relation to the baseline can be considered, as previously discussed about the geochemical indices.

The ecotoxicological indices were in accordance with the sediment quality guidelines, which showed that the concentrations corresponded to the limits determined by CONAMA [46], and the metals Zn, Cu, Cd, Pb and Ni had concentrations below the determined limits.

Exceptions to the sediment quality guideline were verified for Cr, which did not have concentrations below the minimum limit, and for Ni, in which all concentrations were above the determined limits, although the ecotoxicological results of these two metals corresponded to the two lowest classification categories.

In the fish farming area in Changshow Lake, China, Xie et al. [18] found that the metals Zn, Fe, Mn, Cu, Ni, Cr, Se, Hg and Pb in the sediment had concentrations above the background value, but the risk ecological risk to the environment was classified as moderate and the potential ecological risk was higher in the area of fish farming.

The results pointed out by Xie et al. [18] were similar to the data obtained in the Rosário reservoir, as the concentrations were higher than the reference value and the baseline and part of the indices were higher in the cultivation area, but most of the results were higher in the postcultivation area.

Regarding the ecological risk, potential ecological risk and toxic risk index, it was observed that between the PCTV and CTV areas, there was no significant difference; however, the CTRL area was significantly different from the PCTV and CTV areas.

Regarding Lake Baiyangdian, Ji et al. [89] found that the values of $E_{j}^{i}$ for Cd were superior to the other analyzed metals. In the sediment of the Rosário reservoir, this discrepancy of Cd in relation to the other analyzed metals was also verified. This fact probably occured as a function of the cadmium toxic response coefficient, defined by Hankansom [54], considering that the coefficient of Cd is superior to that of other metals due to Cd toxicity. However, the values of $E_{j}^{i}Cd$ obtained from the Rosário reservoir sediment still corresponded to the second risk class.

In the Tishgo and Chia-Peru Rivers, Custódio et al. [51] classified the sediment with potential risk from low to moderate. In Changshou Reservoir, China, the $PERI_{j}$ was classified as moderate [18].

Kanda et al. [50] investigated the ecological risks and potential ecological risks of the Magobo Reservoir, Zimbabwe, and found that the ecological risk was less than 40 and the potential ecological risk was less than 150, and therefore was moderate.

Baiyangdian Lake is a mesotrophic lake, located in the center of a new urban development area in China, and has received effluents from human activities [89]. In this environment, these authors identified the following conditions: ecological risk index $E_{j}^{i}$ < 40, $PERI_{j}$ > 150: high risk, ranging up to $PERI_{j}$ > 560; extremely strong risk: TRIi>5. In some places Cd, Pb and Zn influenced the TRI to be severely polluted.

For decades, geochemical and ecotoxicological indices have been used to determine the level of environmental impact caused by human activities on the sediment. The use of these indices for the evaluation of fish farming in net cages is more recent and, in most cases, they deal with marine and estuarine fish farming. Studies on fish farming in lakes and reservoirs are rare.

In the Qinzhou Bay fish farming area, the IRE varied seasonally; in spring ($E_{j}^{i}\;$= 60) and in the winter ($E_{j}^{i}\;$= 44), both were classified as a moderate ecological risk [78].

In studies carried out in China by Zhang et al. [9] in Hailing-China Bay and Gu et al. [45] in Baisha Bay, areas occupied by fish farming resulted in a low risk for the analyzed metals, except for Cd, which resulted in a strong ecological risk. 

Zhang et al. [9], in Hailing Bay, China, classified the $PERI_{j}$ of the sediment between low, moderate and strong.

In Lake Changshow, the ranking of potential ecological risk values related to fish farming was determined in the following order: Pb > Ni > Cu > Cr > Zn > Mn [18].

The method results used in this study of sediment from the Rosário reservoir are consistent with the study by Nawrot et al. [90]. These authors state that data from the Web of Science Core Collection indicate the highest frequency of surface sediment studies in layers between 0–5 cm to 0–10 cm, with geochemical indices Igeo, EF and CF, as well as with ecotoxicological evaluation using SQG ePERI.

The results obtained in the cluster and principal components analyses showed the influence of organic matter on the trace metals, indicating that the strongest and most significant correlations occurred between organic matter and trace metals, as well as suggesting that the special distribution evidenced the increase in organic matter and metals from the fish farming and postculture areas in relation to the control area, which in most cases were significantly different from the other areas.

Based on the results obtained in this study, we can infer that the use of multivariate statistical techniques, such as hierarchical cluster analysis and PCA, when associated with the use of geochemical and ecotoxicological indices, can efficiently respond to the monitoring of fish farming activities in net cages.

This possibility should be adopted especially in regions where there are no specific monitoring programs for the activity, thus making monitoring similar to what was proposed by Chou et al. [88], who indicated the use of multivariate (PCA and AHC) statistical techniques to environmentally monitor the activity.

## 4. Conclusions

The highest concentrations of Mn, Zn, Cr and Cd occurred in the fish farming area (CTV). Fe, Cu, Ni and Pb had the highest concentrations in the postculture area (PCTV). These two areas were statistically equal but were significantly different from the control area.

The correlation between trace metals, sediment and organic matter indicated a significant correlation between organic matter and the trace metals Mn, Zn, Cu, Cr, Cd, except for Fe and Ni. It also showed that the strongest and most significant correlations between the metals occurred with the main metals in the tilapia feed, Mn/Zn, Cu/Cr, Mn/Cu, Mn/Cr, Zn/Cu and Zn/Cr. These results show the influence of fish farming on the sediment.

The multivariate statistical analysis confirmed the results of the spatial distribution of metals in the sediment and the influence of fish farming on the biogenic action of the sediment, promoted by the residual organic matter of the culture.

The geochemical indices (Igeo, FE, FC and CD) and ecotoxicological indices ($E_{j}^{i}$; $PERI_{j}\;e\;TRI_{i}$) presented results with low environmental risk values; in general, they were classified between the first and second contamination category, which corresponded to the lowest classification levels of the indices.

The sediment quality guideline and the comparison with Resolution 454/2012-CONAMA showed that most of the concentrations verified were within the established limits and were in accordance with international standards. Only nickel had concentrations above the limit established in the DQSe/CONAMA.

From the above, we can say that the twelve years of fish farming in the Rosário reservoir did not promote large-scale changes in the sediment, as the geochemical and ecotoxicological condition suffered minimal changes. Therefore, it is important that further studies be carried out in this line of research, as well as efforts to implement and improve good management practices, mainly to minimize the possibilities of impact on the sediment.

## Figures and Tables

**Table 1:** Quality assurance and quality control procedures for chemical tests, including concentrations in reference samples. NIST 1646 ^a^.

Heavy Metal	Certified Values ^a^	Detection Limits	Observed Concentration	Recovery (%)
Fe ^b^	2.008 ± 0.039	0.12	1.98 ± 0.06	99
Mn ^c^	234.5 ± 2.8	0.10	215.70 ± 4.90	92
Zn ^c^	48.9 ± 1.6	0.26	46.90 ± 2.70	96
Cu ^c^	10.01 ± 0.34	0.08	9.41 ± 0.25	94
Cd	0.148± 0.007	0.03	0.14± 0.01	95
Cr	40.9 ± 1.9	0.10	36.40± 2.50	89
Pb	11.7 ± 1.2	0.10	10.40± 0.90	89
Ni ^d^	23	0.03	21.20± 1.10	92

Standard Reference Material^®^ 1646. ^a^ = Estuarine Sediment; ^b^ = mass percentage (wt%); ^c^ = concentration (mg kg^−1^); ^d^ = noncertified Value.

**Table 2:** Geochemical indices for assessing contamination by trace metals.

Individual Indices	Classification
Geoaccumulation index [52]. $Igeo=\log_{2}\;\left[\;\frac{\left(C^{i}a\right)}{1.5\;X\;\left(C^{i}b\right)}\right]$ Where: $\left(C^{i}a\right)$ = Metal concentration. $(C^{i}b$) = Background value. 1.5 = Minimizing factor of possible background value variation.	Value/category
	<0: Unpolluted; 0–1: Unpolluted to moderately polluted; 1–2: Moderately polluted; 2–3: Moderately to heavily polluted; 3–4: Heavily polluted; 4–5: Heavily to extremely polluted; >5: Extremely polluted.
Enrichment factor [53]. $FE=\frac{\left[\left(C^{i}a\right)/\;\left(C^{i}b\right)\right]}{\left[\left(Fe^{i}a\right)/\;\left(Fe^{i}b\right)\right]}$ Where: $\left(C^{i}a\right)$ = Metal concentration. $\left(C^{i}b\right)$ = Background concentration. $\left(Fe^{i}a\right)$ = Metal concentration. $\left(Fe^{i}b\right)$ = Background concentration.	Value/category
	<1: No enrichment; 1–3: Minimum enrichment; 3–5: Moderate enrichment; 5–10: Moderately severe enrichment; 10–25: Severe enrichment; 25–50: Very severe enrichment; >50: Extremely severe enrichment.
Contamination factor [54]. $CF_{j}^{i}=$ ($C^{i}a)/\left(C^{i}b\right)$ Where: $CF_{j}^{i}=$ individual by metal (*i*) $\left(C^{i}a\right)$ = Metal concentration. $\left(C^{i}b\right)$ = Background concentration.	Value/category
	<1: Low contamination; 1 ≤ FC < 3: Moderate contamination; 3 ≤ FC < 6: Considerable contamination; CF > 6: Very high contamination.
Complex index.	Classification
degree of contamination [54]. $CD_{j}=\overset{n}{\underset{i=1}{\sum }}CF_{j}^{i}$ *CD_j_* = Total metals in a sampling area (*j*). $CF_{j}^{i}$ = Contamination factor.	Value/category
	CD < 6: Low degree of contamination; 6 ≤ CD < 12: Moderate degree of contamination; 12≤ CD < 24: Considerable degree of contamination; CD > 24: High degree of contamination.

**Table 3:** Ecotoxicological indices of metal pollution analysis: ecological risks.

Complex Index	Classification		
Ecological risk [54]. $E_{j}^{i}$ = $T_{c}\;X\;CF_{j}^{i}$ $E_{j}^{i}$ = Index of potential ecological risk of a single heavy metal (i) at the sampling site (j). TC = Heavy metal toxic response coefficient (i) = (Zn = 1, Cu = 5, Cr = 2, Cd = 30, Pb and Ni = 5).	Value/Category		
	1-$E_{j}^{i}$ < 40: low ecological risk; 2–40 <$\;E_{j}^{i}$ < 80: moderate; 3–80 <$\;E_{j}^{i}$ < 160: strong; 4–160 <$\;E_{j}^{i}$ < 320: very strong; 5-$E_{j}^{i}\;$> 320: extremely strong.		
Potential ecological risk [56]. ${\mathrm{PERI}}_{j}=\overset{n}{\underset{i=1}{\sum }}E_{j}^{i}$ ${\mathrm{PERI}}_{j}$ = Comprehensive potential ecological risk at the sampling site (j).	Ecological risks		
	1-${\mathrm{PERI}}_{j}$ < 70: low; 2–70 $<{\mathrm{PERI}}_{j\;}$< 140: moderate; 3–140 $<{\mathrm{PERI}}_{j\;}$< 280: strong; 4–280 $<{\mathrm{PERI}}_{j}$ < 560: very strong; 5-$<{\mathrm{PERI}}_{j}$ > 560: extremely strong.		
Toxic risk index [9]. TRIi = $\sqrt{({\left(C^{i}/TEL\right)}^{2}+{\left(C^{i}/PEL\right)}^{2})/2}$ TRIi = toxic risk index $C^{i}$ = metal concentration in the sediment TEL *: threshold effect level ***; PEL **: likely effect level ***;	Classification		
	Value/category		
	1-TRI ≤ 5: no toxic risk; 2–5 < TRI ≤ 10: low toxic risk; 3–10< TRI ≤ 15: moderate toxic risk; 4–15 < TRI ≤ 20: considerable toxic risk; 5-TRI > 20: very high toxic risk.		
Sediment Quality Guidelines [46]	Metal	Concentration (mg/kg^−1^)	
		Level I	Level II
	Zn	123	315
	Cu	35.7	197
	Cr	37.3	90
	Cd	0.6	3.5
	Pb	35	91.3
	Ni	18	35.9

* = Level I (NI) Resolution 454/2012-CONAMA; ** = Level II (NII) Resolution 454/2012-CONAMA and *** (CCME/Long and MacDonald, 1998).

**Table 4:** Mean granulometry of the sediment in the study areas.

Area	Sand	Clay	Silt
PCTV	12.70 ± 6.00	35.49 ± 22.35	51.81 ± 26.48
CTV	21.32 ± 8.35	30.04 ± 22.66	51.33 ± 23.10
CTRL	9.41 ± 8.35	40.11 ± 20.60	48.47 ± 22.68

**Table 5:** Concentrations of trace metals in the superficial sediment of the Rosário reservoir and values of sediment quality guidelines (SQGs).

	PCTV		CTV		CTRL		SQGs *	
	(Non-Fish-Farming Area)		(Fish Farming)		(Control Area)			
	Min/Max	Average/σ	Min/Max	Average/σ	Min/Max	Average/σ	Level N1	Level N2
Fe ^a^	0.13	0.36 ± 0.14	0.19	0.40 ± 0.22	0.11	0.28 ± 0.14	-	-
	0.59		0.85		0.51			
Mn ^b^	38.3	51.93 ± 8.49	27.88	66.48 ± 19.11	14.46	22.55 ± 4.75	-	-
	64.51		86.91		28.56			
Zn ^b^	29.3	77.77 ± 50.98	22.79	114.83 ± 59.75	2.6	9.13 ± 3.80	123	315
	187.09		220.56		15.33			
Cu ^b^	29.1	71.83 ± 21.20	40.46	62.23 ± 18.57	26.89	37.74 ± 8.96	35.7	197
	98.75		91.3		48.3			
Cr ^b^	65.06	70.53 ± 3.54	68.21	70.85 ± 2.62	60.18	66.45 ± 4.21	37.3	90
	76.06		74.69		74.13			
Cd ^b^	0.46	0.53 ± 0.04	0.4	0.47 ± 0.04	0.38	0.43 ± 0.03	0.6	3.5
	0.59		0.53		0.46			
Pb ^b^	29.5	33.71 ± 4.34	23.13	26.29 ± 2.32	18.13	18.77 ± 0.59	35	91.3
	43.13		29.75		19.75			
Ni ^b^	41.26	44.60 ± 1.79	36.45	40.99 ± 2.43	34.4	39.17 ± 3.76	18	39
	46.75		45.8		44.81			

σ = standard deviation; ^a^ = (%); ^b^ = (mg/kg^−1^). * SQGs = S\sediment quality guidelines CONAMA [46].

**Table 6:** Increment of metals in the CTV and PCTV areas in relation to the control area.

	Concentrations	Percentage Increases	
Metals	CTRL	CTV	PCTV
Fe (%)	0.28 ± 0.14	30%	34.88%
Mn (mg/kg^−1^)	22.55 ± 4.75	66.08%	59.66%
Zn (mg/kg^−1^)	9.13 ± 3.80	92.05%	90.32%
Cu (mg/kg^−1^)	37.74 ± 8.96	39.35%	47.46%
Cr (mg/kg^−1^)	66.45 ± 4.21	6.21%	5.78%
Pb (mg/kg^−1^)	18.77 ± 0.59	28.60%	47.69%
Ni (mg/kg^−1^)	39.17 ± 3.76	4.44%	12.17%

**Table 7:** Correlation between metals and sediment from the Rosário reservoir.

	Fe	Mn	Zn	Cu	Cr	Cd	Ni	<63 μm	M.O.
Fe									
Mn	0.29 ^ns^								
Zn	0.41 **	0.76 *							
Cu	0.21 ^ns^	0.62 *	0.56 *						
Cr	0.20 **	0.58 *	0.51 *	0.68 *					
Cd	0.18 ^ns^	−0.33 ^ns^	−0.34 ^ns^	−0.51 *	−0.34 ^ns^				
Ni	0.18 ^ns^	0.13 ^ns^	0.07 ^ns^	0.48 *	0.47 **	−0.50 *			
<63 μm	0.01 ^ns^	−0.02 ^ns^	0.09 ^ns^	−0.07 ^ns^	0.04 ^ns^	0.08 ^ns^	0.01 ^ns^		
M.O.	0.04 ^ns^	0.65 ^ns^	0.46 **	0.46 *	0.40 **	−0.41 **	0.13 ^ns^	0.36 ^ns^	

* (*p*<0.05), ** (*p*<0.01) and ^ns^ = not significant.

## Data Availability

The data are available upon official request from the principal investigator.
